# Psychotropic Medication Trends Before, After Suicide Attempt Among Mid-To Late Life US Adults With Bipolar Disorder

**DOI:** 10.1093/geroni/igaf122.2710

**Published:** 2025-12-31

**Authors:** Randall Kuffel, Ramin Mojtabai, Michael Steinman, Matthew Growdon, Yixia Li, John Boscardin, G Caleb Alexander, Amy Byers

**Affiliations:** Johns Hopkins Bloomberg School of Public Health, Baltimore, Maryland, United States; Tulane University School of Medicine, New Orleans, Louisiana, United States; University of California, San Francisco, San Francisco, California, United States; University of California, San Francisco, San Francisco, California, United States; Northern California Institute for Research and Education, San Francisco, California, United States; University of California, San Francisco, San Francisco, California, United States; Johns Hopkins Bloomberg School of Public Health, Baltimore, Maryland, United States; University of California, San Francisco / SFVAHCS, San Francisco, California, United States

## Abstract

It is unclear how medication regimens change following a non-fatal suicide attempt in mid-to late life adults with bipolar disorder. We characterized psychotropic medication use among these patients 1 year before and after a suicide attempt. Using a retrospective cohort of US veterans ages 50 and older with bipolar disorder, we matched patients attempting suicide between October 2011 and December 2019 to similar patients who did not attempt suicide. We measured quantity of fills and prevalence of use for antidepressants, antiepileptics, antipsychotics, benzodiazepines, lithium, opioids, sedative hypnotics, and polypharmacy in the 4 quarters before and after suicide attempt. Our final sample included 4,747 patients who attempted suicide and 4,747 matched controls. Mean age at index was 60 and 86% were male. At baseline, individuals attempting suicide were treated with antidepressants (60.0%), antiepileptics (48.0%), antipsychotics (41.2%), benzodiazepines (27.1%), lithium (5.4%), opioids (32.8%), and sedative hypnotics (14.9%). Many patients were on three or more psychotropic therapy classes at baseline (overall 30.8%). Psychotropic medication use was consistently greater among those attempting suicide than their matched counterparts for all medication classes except opioids. After their suicide attempt, there were large and abrupt increases in antiepileptics, antipsychotics, and sedative hypnotics, with smaller increases in antidepressants, benzodiazepines, and lithium, and reductions in opioids. These differences persisted up to six months following suicide attempt. Findings indicate patients and clinicians may make comprehensive changes to treatment plans in the short and long term. Further research is needed to understand the impact of these changes on future suicide risk.

